# The impact of pharmacist practice of medication therapy management in ambulatory care: an experience from a comprehensive Chinese hospital

**DOI:** 10.1186/s12913-023-09164-6

**Published:** 2023-02-21

**Authors:** Qingli Meng, Lulu Sun, Yingjie Ma, Yuanyuan Wei, Xiaowei Ma, Lu Yang, Zhengzheng Xie, Fang Li, Zhe Wang, Xiaomei Tao, Xia Zhen, Rui Jin, Hongyan Gu

**Affiliations:** 1 Department of Pharmacy, Beijing Shijitan Hospital, Capital Medical University, Beijing, 100038 China; 2 Beijing Key Laboratory of Bio-Characteristic Profiling for Evaluation of Rational Drug Use, Beijing, 100038 China; 3 International Cooperation & Joint Laboratory of Bio-Characteristic Profiling for Evaluation of Rational Drug Use, Beijing, 100038 China

**Keywords:** Medication therapy management services, MTMs, Pharmaceutical care, Rational use of drugs

## Abstract

**Backgroud:**

With the reform of medical system in China, Beijing municipal hospitals explored a new pharmaceutical care model and set up medication therapy management services (MTMs) in ambulatory care since 2019. We were one of the first hospitals to set up this service in China. At the present, there were relatively few reports about the effect of MTMs in China. In this study, we summarized the implementation of MTMs in our hospital, explore the feasibility of pharmacist-led MTMs in ambulatory care and the impact of MTMs on patients’ medical costs.

**Methods:**

A retrospective study was conducted in a university-affiliated, tertiary comprehensive hospital in Beijing, China. The patients who received at least one MTMs and with complete medical records and pharmaceutical documents from May 2019 to February 2020 were included. Pharmacists provided pharmaceutical care for patients according to the MTMs standards issued by the American Pharmacists Association, identified the numbers and classification of the patients’ perceived medication-related demands, identified medication-related problems (MRPs), and developed the medication-related action plans (MAPs). All MRPs found by pharmacists, pharmaceutical interventions, and resolving recommendations were documented, and calculate the cost of treatment drugs that patients can reduce.

**Results:**

A total of 112 patients received MTMs in ambulatory care, among them 81 cases with the completed record were included in this study. 67.9% of patients had five or more diseases, 83% of them co-took over 5 drugs. While performing MTMs, 128 patients’ perceived medication-related demands were recorded in all, monitoring and judgment of adverse drug reaction (ADR) (17.19%) was the most common demand. 181 MRPs were found, with an average of 2.55 MPRs per patient. Nonadherence (38%), excessive drug treatment (20%), and adverse drug events (17.12%) were the top three MRPs. Pharmaceutical care (29.77%), adjustment of drug treatment plan (29.10%) and referral to the clinical department (23.41%) were the top three MAPs. Whereby the MTMs provided by pharmacists, the cost-saving of each patient was about $ 43.2 monthly.

**Conclusion:**

By participating in the MTMs of outpatients, the pharmacists could identify more MRPs and develop personalized MAPs timely for patients, thereby promoting rational drug use and reducing medical expenses.

**Supplementary Information:**

The online version contains supplementary material available at 10.1186/s12913-023-09164-6.

## Background

Medication therapy management (MTM) refers to broad services provided by professional pharmacists to optimize therapeutic outcomes for patients, such as medication instruction, consulting, education, and guidance. MTM includes 5 components: medication therapy review (MTR), personal medication record (PMR), medication-related action plan (MAP), intervention and/or referral, and documentation and follow-up. Studies have shown that MTM help improve medication adherence rates, improve medication safety, effectiveness, and reduce the occurrence of medication errors [1]. Although the term “MTM” was used in 2003, MTM similar services were provided by the pharmacist as early as the 1990s in the United States. The long-term practice has proved that this service program is beneficial as it can reduce the economic burden of the patients, in addition to promoting the medications rationally [2–6]. Currently, it has been approved by the current medical insurance laws and has taken a significant role in American medical services.

In the 1950s, medical institutions in China used high profits of drugs to promote the economic benefits of hospitals and maintain the normal operation of hospitals. However, this mode of operation eventually led to the phenomenon of expensive and difficult medical care. In order to improve this phenomenon, China implemented the medical system reform policy in 2009, vigorously promoted the separation of medicine system, cut off the direct economic interests between medical institutions and drug marketing one by one, and reduced the burden of patients’ medical care. In this reform, the working mode of pharmacists was also changed from “drug-centered” to “patient-centered”. In order to meet the needs of medical system reform and ensure the best therapeutic outcomes for patients, Beijing Pharmacists Association (BPA) firstly cooperated with American Pharmacists Association (APhA) to introduce MTM in China and successfully held three training courses in 2016. In 2017, MTM training program was redesigned and localized in Beijing by Beijing Hospital Authority in cooperation with BPA [7]. The municipal hospitals were encouraged to provide medication therapy management services (MTMs) in ambulatory care settings as well as outpatient settings. Until May 2019, MTMs were implemented in at least 22 hospitals. However, there are few articles on the evaluation of the benefits to patients after the implementation of MTMs, such as health benefits or economic benefits. In this study, we summarized the implementation of MTMs in our hospital and explore the impact of MTMs on rational, safe, and economical use of drugs.

This study aimed to assess the beneficial effect of pharmacist-led MTMs on patients and discuss the current status and challenges of pharmacist-led MTMs in China.

## Methods

### Study design

We chose Beijing Shijitan Hospital as the site of the retrospective study because it is a tertiary general hospital with an average daily outpatient flow of 2000, and the elderly are the main patient group. Consecutive patients were enrolled from May 2019 to February 2020. Thirteen clinical pharmacists interviewed patients at the pharmacist outpatient clinic. Ten pharmacists had completed training program accredited by the APhA. One pharmacist had completed MTM training program accredited by Beijing Hospital Authority and BPA. The other two clinical pharmacist had more than 5 years of hospital practice experience.

### Patient eligibility and workflow

**Fig. 1:**
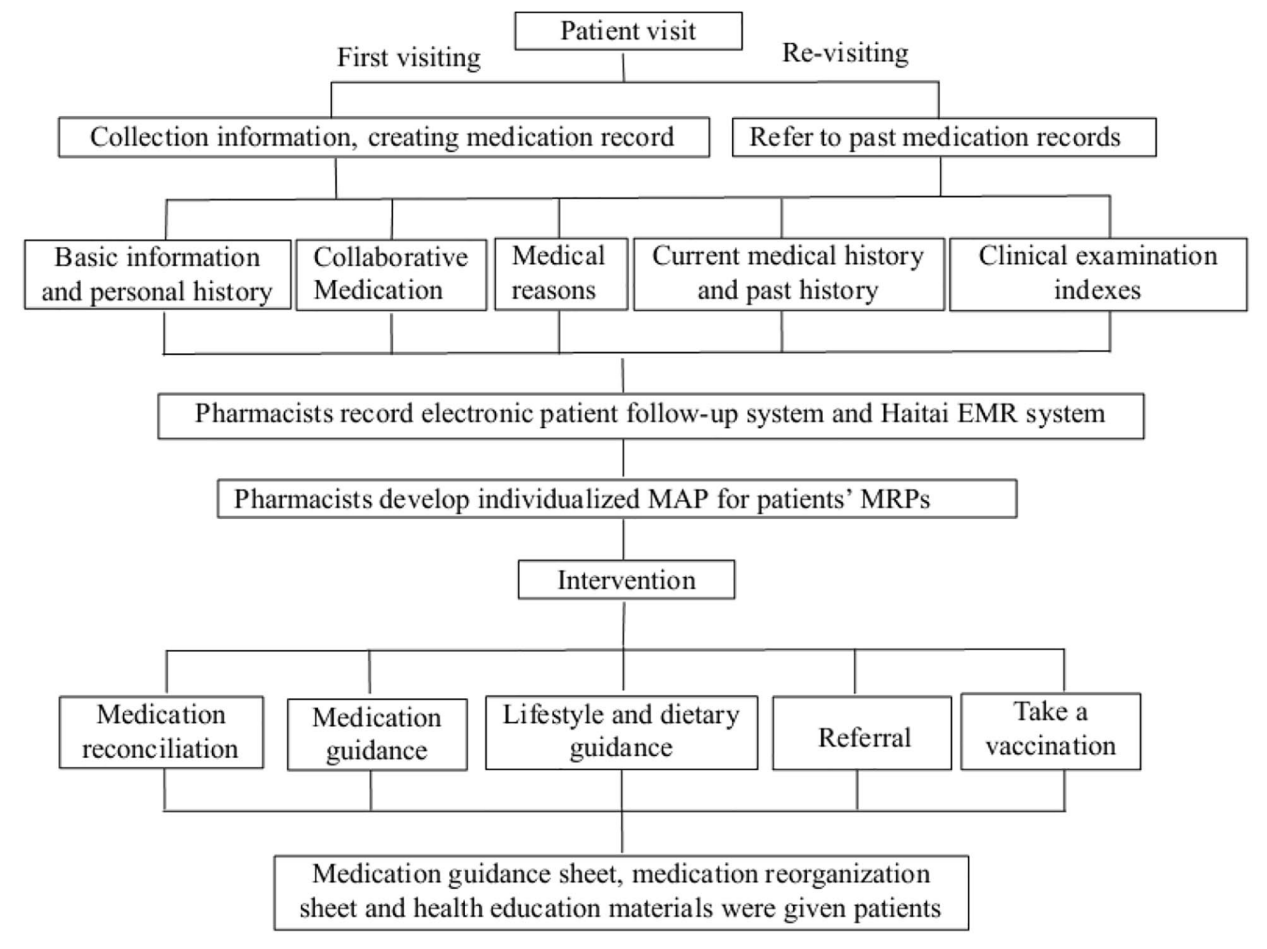
MTM pharmacy clinic service workflow

Patients meeting the following conditions will be included in this study:1) received at least one pharmaceutical care at MTM ambulatory care, 2) with complete medical records and pharmaceutical documents in the electronic patient follow-up system and Haitai electronic medical record (EMR) system in our hospital. Haitai EMR system is a software used by doctors to record patients’ medical information, including the patient’s name, gender, chief complaint, present history, past medical history, diagnosis, prescription drugs and the usage of taking the medicine, etc. On the contrary, patients with incomplete medical records and pharmaceutical documents were excluded. The MTMs process followed the work process recommended by APhA, such as collected clinical and medication information, filled in PMR, performed documentation, identified medication-related problems (MRPs), provide MAP, and performed follow-up, Fig. 1 displays the MTMs workflow [8].

### Study measurements

Baseline demographic information included patient age, sex, comorbidities, the number of combined drugs, and relevant clinical or laboratory examination indexes were collected. Record the generic name, preparation type and the cost of the drugs used by the patients.

The rationality of drug therapy was evaluated according to the latest domestic treatment guidelines [9–17]. The MRPs were classified into seven categories according to Pharmacy Administration Commision of Chinese Hospital Association classification standard, including excessive drug treatment, inadequate medication regimen, ineffective medication, insufficient drug dosage, adverse drug event, dosage too high and non-adherence [18]. Details of each category are explained in supplementary material table S1. Medication-related action plans (MAPs) were identified in corresponding to the MRPs by the service pharmacists.

In this study, chemical synthetic drugs are classified according to pharmacological effects. Chinese patent medicines are classified according to drug efficacy, which is the classification method used in the basic drug catalog of Chinese patent medicines.

The acceptance rate of MTMs intervention is one of the outcome indicators of this study. Pharmacists calculated the acceptance rate of pharmaceutical care intervention by consulting the diagnosis and treatment information of patients in other clinical departments.

The cost of drugs saved by pharmaceutical outpatient service for patients is another outcome indicator of this study. In this study, self-control method was adopted to calculate this index. First, calculate the average monthly drug cost of the patient before going to the MTM pharmacy clinic and after receiving MTMs [19], and then calculate the difference between the two, which is the average monthly drug cost saved for the patient. Since pharmacists cannot restrict patients to select drug manufacturers and test levels, this study only calculates the cost of treatment drugs that patients need to save. The number of prescription days in a month is 28 days.

### Statistical analysis

Descriptive statistics were used to characterize the study group. Continuous variables were described as means ± SDs. Categorical variables were described as percentage distributions. SPSS version 23.0 software is used to analyze continuous data. A P value of < 0.05 was considered to be statistically significant.

## Results

### Baseline characteristics

The 10-month evaluation from May 2019 to February 2020 was conducted and 112 enrollees visited the MTM ambulatory care. Of these patients, 81 patients were identified with medical records fully reviewed by the pharmacists of MTM ambulatory care. Their basic characteristics were summarized in Table 1.

Among the patients, two patients were younger than 40 years old, and others were older than 40 years old. Patients over 65 years old accounted for 71.6%, which was the main visiting population. The visit rate of female was higher than male. One patient had one disease, the others were all multimorbidity. 55 patients had five or more diseases, accounting for 67.90%. Hyperlipidemia, coronary heart disease, hypertension were the majority comorbidities. 44.44% of the 81 patients have taken 5 to 9 medications and 38.27% have taken over 10 medications. Four patients (4.94%) indicated a history of using health supplements.

**Table 1:** Baseline Characteristics of patients included in this study

Characteristics	No.
Age, n (%)	
< 40	2 (2.47)
40–64	21 (25.93)
65–79	34 (41.98)
≥ 80	24 (29.63)
mean	71.05 ± 13.01
Sex, n (%)	
male	33 (40.74)
female	48 (59.26)
Number of comorbidities, n (%)	
1	1(1.23)
≥ 2 and <5	25(30.86)
≥ 5	55 (67.90)
Types of comorbidities*, n (%)	
Hyperlipidemia	54 (66.67)
Coronary heart disease	32 (39.51)
Hypertension	32 (39.51)
Cerebral infarction	21 (25.93)
Diabetes	17 (20.99)
Hyperuricemia / gout	12 (14.81)
Sleep disorders	11 (13.58)
Arthritis	11 (13.58)
Osteoporosis	11 (13.58)
Peripheral neuropathy	10 (12.35)
Number of combined drugs, n (%)	
1–4	14 (17.28)
5–9	36 (44.44)
≥ 10	31 (38.27)

*: Due to the large number of diseases involved, only diseases with more than 10 persons were listed

Note: Data are mean ± SD or n (%)

### Medication-related problems

**Fig. 2:**
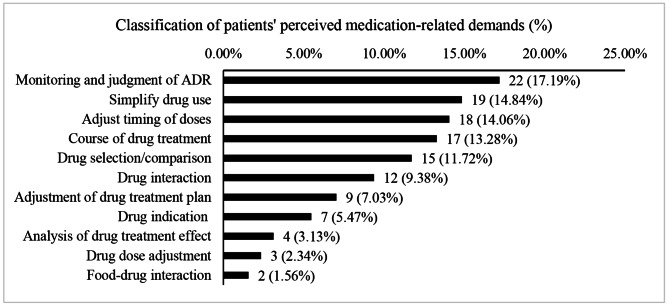
Classification of patients’ perceived medication-related demands

128 patients’ perceived medication-related demands were recorded. Among them, monitoring and judgment of adverse drug reaction (ADR) was the main problem, accounting for 17.19%, followed by simplifying drug varieties (14.84%) and adjusting the timing of doses (14.06%). The corresponding results were shown in Fig. 2.

Among 81 patients, 87.65% (n = 71) patients were identified with MRPs. 181 MRPs were found, with an average of 2.55 MRPs per person. Nonadherence, excessive drug treatment, and adverse drug event were the main MRPs, accounting for 38%, 20%, and 17.12% respectively. The classification and causes of MRPs were summarized in Table 2.

That patients did not understand the instructions was the most common reason for nonadherence, accounting for 80.88% (n = 55). The main clinical manifestations were as follows: (1) taking proton-pump inhibitors (PPIs) for more than 6 months to prevent ulcer/gastrointestinal bleeding reaction, (2) taking aspirin enteric-coated tablets after meals due to stomach discomfort, (3) taking levothyroxine sodium and milk together, (4) taking sustained or controlled release preparation by sublingual, (5) reducing the frequency of taking medicines due to worrying about ADR. Except that, some patients did not receive antiplatelet therapy and lipid-lowering treatment for secondary prevention of coronary artery disease. 14.7% (n = 10) patients were unwilling to take drugs owing to fear of ADR, which was the second reason for nonadherence.

Among the excessive drug treatment problems, 47.22% (n = 12) of the patients took multiple drugs to treat one disease, 38.89% (n = 10) had no indication. Drugs that need to stop taking include chemically synthesized drugs, Chinese patent medicine and dietary supplements. The main problems with the treatment of chemically synthetic drugs were taking PPIs without indication, taking folic acid and vitamin B for a long time after the cure of hyperhomocysteinemia. The problems of taking Chinese patent medicines include taking different compositions but same efficacy medicines, taking same main components and same efficacy medicines, or taking medicines that were not recommended by the guidelines or have adverse interactions with the current therapeutic drugs. Blood-regulating recipes (promoting blood circulation and removing blood stasis) was the main drug involved. In this study, 25 patients would reduce their medical expenses by simplifying their prescription.

**Table 2:** Classification of MRPs

Classification	%
Excessive drug treatment	
No indication of medication	7.73
Excessive combination therapy	9.39
No medication required	2.76
Inadequate medication regimen	
Need to start new drugs to treat new diseases or complications	9.94
Preventive medication therapy is required to reduce the risk of developing a new condition	1.10
Additional drugs are needed to obtain synergistic or adjunctive therapeutic effects.	2.76
Ineffective medication	
The selected drugs are not the best treatment for patients’ diseases	4.42
Insufficient drug dosage	
Dosage too low	2.76
Drug interactions weaken the effective drug dosage	1.10
Adverse drug event	
Non-dose-dependent adverse drug reactions	15.47
Safer drugs need to be chosen because of the risk factors	1.10
Medication is contraindicated because of risk factors	0.55
Dosage too high	
Single dose too high	2.21
Duration of medication therapy is too long	1.10
Nonadherence	
Patients do not understand the medication instructions and guide	30.39
Subjectively unwilling to take medicine	5.52
Patient forgets to take medication	1.66

### Contents of MAPs

For each MRP detected in the MTMs, pharmacists provided 299 individualized MAPs in all and completed 586 medication reconciliation and medication education. The contents of MAPs can be summarized into five categories, including pharmaceutical care, adjustment of the drug treatment plan, referral to the clinical department, diet and exercise suggestions, and others. The specific content and proportion of each classification were shown in Table 3. Pharmaceutical care, adjustment of the drug treatment plan, and referral to the clinical department were the top three MAPs, accounting for 29.77%, 29.10%, and 23.41% respectively.

**Table 3:** Classification of MAPs

Classification	%
Pharmaceutical care (adverse reactions/efficacy/others)	29.77
Adjustment of drug treatment plan	
Reduce the variety of treatment drugs / discontinuation	13.38
Medication time adjustment	6.02
Change of therapeutic drugs	5.35
Increase the variety of therapeutic drugs	2.68
Dosage adjustment	1.00
Adjustment of medication method	0.67
Referral to clinical department	23.41
Diet and exercise suggestions	16.39
Others	
Vaccination	0.67
Emergency medication	0.33
Storage of drugs when outside of home	0.33

### Outcome of MTM pharmaceutical care intervention

41 of the 81 patients received 87 recommendations to adjust their treatment regimen. Eleven of them fell out and did not come to the hospital again after this pharmaceutical care. For the remaining 30 patients, there were 54 suggestions to adjust the drug therapy regimen, of which 40 were accepted by the patients, with a total acceptance rate of 74.07%. Among the patients who adjusted their medication regimens, one patient showed no improvement in insomnia after zolpidem was changed to zopiclone. One month later, the patient went to the outpatient again and was prescribed zolpidem and estazolam for treatment. Through checking the records of the outpatient EMR system of Haitai, it was not found that these patients used the simplified drugs again in the next six months.

### Cost-saving of pharmaceutical care

**Fig. 3:**
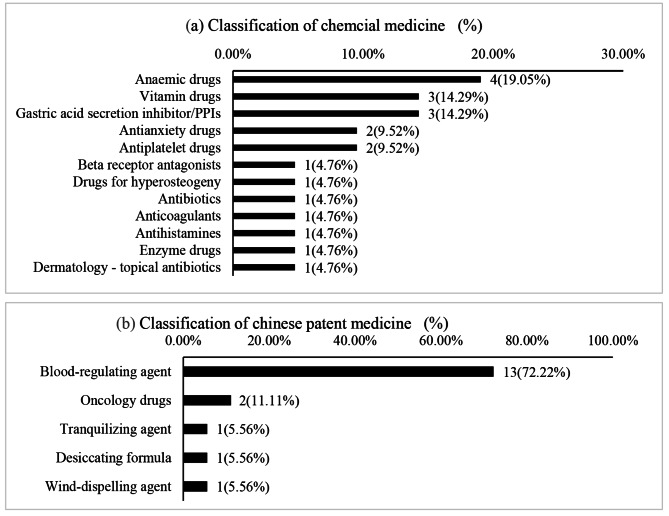
Classification of drugs to be discontinued

Through pharmaceutical outpatient service, pharmacists successfully intervened in 25 patients to stop 42 drugs and dietary supplements, of which 50% were chemical synthetic drugs, 47.62% were Chinese patent drugs, and 2.38% were dietary supplements. The chemically synthesized drugs can be divided into 12 categories according to the mechanism of action, and Chinese patent drugs can be divided into 5 categories according to the efficacy of drugs. The corresponding results were shown in Fig. 3. Anti-anemia drugs, vitamin drugs, and gastric acid secretion inhibitor/ PPIs were the top three in chemical medicines. According to the classification of drug effect, blood-regulating agent was the largest proportion in Chinese patent medicines, involving drugs such as wenxinkeli, sanqi tongshu capsules and so on. Adjuvant drugs for tumor account for the second, bailing capsule was the only medicine involved. Sedative tranquillizing formula, desiccating formula and dispel-wind drugs were next. The drugs involved were qingnao fushen oral liquid, qianlie shutong capsule and tianshu capsule. Dietary supplements include liver tonic, multivitamin and Elements Tablets (29-II), tianqu yizhikang tablets.

In our survey, the average monthly cost of drugs per patient dropped from $238.99 to $195.79. 25 patients can save a total of 43.2 dollars per month. The result was showed in Table 4. It implies that the MTM services provided by pharmacists were crucial in preserving medication.

**Table 4:** Average cost of medications per patient for every month

	Pre-intervention	Post-intervention	P
Average drug cost per patient for every month ($)	238.99 ± 128.21	195.79 ± 129.94	< 0.001

## Discussion

### Current status and challenges of MTMs in our hospital

At present, most of the pharmaceutical outpatient clinics in China are targeted at people with specific diseases, such as hypertension patients, diabetes patients, hyperuricemia patients, patients taking anticoagulant drugs, epilepsy patients, etc. [20–22]. As our hospital is a comprehensive tertiary hospital, the pharmaceutical out-patient service was for all patients. In this way, the current needs of patients for pharmaceutical care can be more comprehensively observed. The result of patients’ baseline characteristics showed that patients of any age had the need for pharmaceutical care, which indicates that it was necessary to set up outpatient pharmaceutical care. Due to the rapid development of aging in China [23], elderly patients with comorbidities and multiple drugs were still the main group of pharmaceutical outpatient clinic.

To be able to perform comprehensive pharmaceutical care, the pharmacists may need to spend a great deal of time to collect patients’ disease-related information. In our research, in order to improve the efficiency of pharmaceutical care and shorten the time of collecting information, the pharmaceutical service records were created in the outpatient EMR system. Pharmacists can obtain the patients’ previous information directly by searching their names. The average time for collecting basic information of each patient was about 20 min, and the total length of pharmaceutical care for patients was determined by the number of comorbidity and medication. The average service time of each patient was one hour, which was shorter than previous studies [24].

The total number of MRPs that pharmacists identified was 1.4 times of patients’ concerns, more MRPs ignored by patients can be found by pharmacists. The average number of MRPs per person in our study is slightly higher than that of Wang Xin’s study [19] and Li Yuan’s study [25], but significantly lower than that of Wu Mingfen’s study [24]. The patients included in Wu Mingfen’s study need to meet the following conditions: (1) have more than two chronic diseases; (2) patients who take more than five drugs for chronic diseases at the same time; (3) patients who have ADR and need to adjust treatment drugs; (4) patients taking high-risk drugs with small treatment windows; (5) accept prescriptions from different doctors, etc. These groups of people are more prone to treatment-related problems. Therefore, the average number of MRPs per person was 5.4 in her study [24], which is twice of our study. In the study of Wang Xin [19] and Li Yuan [25], the elderly patients with chronic diseases over 65 years old, the patients with hypertension who did not meet the standard and the patients with multiple drugs were included, respectively. These two articles have less limited criteria for inclusion of patients, so their results are close to the results of this study, the MRP per person was 2.15 [19] and 2.4 [25] respectively. In addition, the PNCE (V8.03 ) classification method for MRP was used in Wang Xin’s research, which only includes 7 types of MRP. This may lead to the lowest result [24].

Monitoring and judgment of ADR was the most direct and easily found problem during the treatment with medicine, and it was also the main MRP that patients paid attention to. However, pharmacists found that the most crucial MRP among patients was medication non-adherence. The possible reason for this phenomenon was that adverse events due to poor adherence usually take longer to occur than adverse drug reactions, patients often paid more attention to the safety of drugs and ignore the importance of medication compliance, especially for chronic patients who need to take drugs for a long time. As we all know, ensuring adequate adherence to the prescribed therapeutic regimen is an important guarantee for the treatment effect and also promotes the prognosis of patients, poor adherence would lead to premature death or a huge financial burden. Hence, improving patients’ medication adherence was one of the major pharmaceutical care of our MTM outpatient service. 80.88% of the medication adherence problems could be attributed to patients not knowing medication information. For example, the patients do not know why to take the drug, the correct use and dosage of the drug, the course of treatment, the interaction between food and drug. This finding means that pharmacists may be able to significantly reduce the MRPs and improve the treatment outcomes of patients by explaining medication knowledge to patients in the MTM ambulatory care.

Reducing the number of drug varieties was the secondary problem of patients, and it was also the second MRP found by pharmacists. Repeated medication is still common in China because each hospital uses an independent local area network system, we cannot obtain the information of patients in other hospitals. Except that the patients’ attention to disease treatment and safe medication does not match their medical knowledge level, the combination of self-medication and professional diagnosis and treatment increases the risk of repeated drug use. This problem further indicated the necessity of establishing pharmaceutical care outpatient clinics and its unique advantages in reducing MRPs.

### Limitations

Although the pharmaceutical care outpatient work flow of our hospital has been constructed and the receiving work was smooth, there are still some limitations in this study.

First, this is a single-centre study, so results and conclusions could be influenced by clinicians’ medication habits, the types of drugs available in hospital, and patients’ disease profiles. Second, this is a retrospective analysis study. Since the contact information of patients was not collected at the beginning of the study, the follow-up work could not be carried out through direct contact with patients, which led to the fact that the acceptance rate of patients to pharmacist’s recommendations could only be analyzed through voluntary return visits of patients or consulting the clinical department records of patients, and patients who did not come back to the hospital would be lost. Third, this investigation was analysis with a limited patient population. As a result, it was unable to do a subgroup analysis according to the type of sickness to obtain more accurate research results. To confirm the current finding and reduce the bias conducting a case-control study is important. At the same time, in order to enable more patients to receive pharmaceutical care, we will publicize pharmaceutical care outpatient to doctors in our hospital, which will help doctors fully understand the role of MTMs and recommend patients to visit. Or we can serve more patients by establishing a combination pharmacy-doctor clinic. Four, as mentioned in the method section, each pharmacist who was involved did thorough drug reviews. However, it is still possible that there are inter-rater reliability constraints. Finally, due to the lack of normal nursing group, this study can only use the self-control method to analyze the savings in drug treatment costs of patients after receiving MTMs. Since pharmacists cannot restrict patients to choose the manufacturer of drugs and examination level, the cost-saving calculation didn’t include the cost of increasing treatment drugs and the increased cost of clinical test. Nevertheless, despite its limitations, this study can still offer some tangible proof that the implementation of pharmacist led MTMs in China has some influence on resolving patient medication issues, promoting rational drug use and improving patients’ drug compliance.

## Conclusion

To sum up, the setting of MTM ambulatory care is conducive for the pharmacists to participate in the treatment and management of outpatients. As a result, pharmacists can discover more MRPs, promote rational drug use, and ensure medical safety and effectiveness, especially for elderly patients with comorbidities and multiple drugs. In addition, professional medication suggestions provided by the pharmacists will increase the patients’ awareness of drugs, improve adherence and reduce the economic burden. However, the MTM ambulatory care is still in the development stage, the workflow needs to be further standardized and patients’ cognition of MTMs needs to be further improved.

## Electronic supplementary material

Below is the link to the electronic supplementary material.

Supplementary Material 1

## Data Availability

The datasets generated and analysed during the current study are not publicly available due privacy but are available from the corresponding author on reasonable request.

## Abbreviations

ADR: adverse drug reaction
APhA: American Pharmacists Association
BPA: Beijing Pharmacists Association
EMR: electronic medical record
MAP: medication-related action plan
MRP: medication-related problem
MTM: medication therapy management
MTMs: medication therapy management services
MTR: medication therapy review
PMR: personal medication record
PPIs: proton-pump inhibitors
