# Supplementary material for: The impact of pharmacist practice of medication therapy management in ambulatory care: an experience from a comprehensive Chinese hospital
Source: BMC Health Serv Res. 2023 Feb 21;23:176. doi: 10.1186/s12913-023-09164-6 (PMC9945368; doi:10.1186/s12913-023-09164-6)
Supplement: Supplementary file 1 — Supplementary Material 1 [file 12913_2023_9164_MOESM1_ESM.docx]

Supplementary material

Table S1 Classification of MRPs

| 1.Excessive drug treatment | Including the following reasons for excessive drug treatment：1) No indication of medication；2) Excessive combination therapy；3) No medication required；4) Use one drug to treat the adverse reaction caused by another drug, which can be solved by other methods. |
| --- | --- |
| 2.Inadequate medication regimen | Including the following reasons for inadequate medication regimen ：1) Need to start new drugs to treat new diseases or complications；2) Preventive medication therapy is required to reduce the risk of developing a new condition；3) Additional drugs are needed to obtain synergistic or adjunctive therapeutic effects. |
| 3.Ineffective medication | Including the following reasons for ineffective medication：1) Patient developed resistance to the drug;2) Improper drug preparation type or route of administration;3) The selected drugs are not the best treatment for patients' diseases. |
| 4.Insufficient drug dosage | Including the following reasons for insufficient drug dose：1) Dosage too low; 2) Too long interval between medication; 3) Drug interactions weaken the effective drug dosage; 4) Drug treatment time is too short |
| 5.Adverse drug event | Including the following situations：1) Non-dose-dependent adverse drug reactions; 2) Safer drugs need to be chosen because of the risk factors; 3) Non-dose-related adverse reactions caused by drug interactions; 4) Adjust medication regimen too quickly; 5) Drug related allergic reactions; 6) Medication is contraindicated because of risk factors; 7) Improper use of dosage or dosage form |
| 6. Dosage too high | Include the following reasons for a drug dose higher than the patient's disease requirement:1) Single dose too high; 2) Interval between medication is too short; 3) Duration of medication therapy is too long; 4) Toxic reaction caused by drug concentration rise due to drug interaction; 5) Excessive speed of administration |
| 7. Nonadherence | Including the following causes of patients' medication compliance problems  1) Patients do not understand the medication instructions and guide; 2) Subjectively unwilling to take medicine; 3) Patients forget to take medication; 4) Patients are economically limited; 5) Unable to take or use drugs by oneself due to physical conditions; 6) Unable to obtain medicine |
